# Protein S-Acyl Transferase GhPAT27 Was Associated with *Verticillium wilt* Resistance in Cotton

**DOI:** 10.3390/plants11202758

**Published:** 2022-10-18

**Authors:** Yahui Deng, Quanjia Chen, Yanying Qu

**Affiliations:** Engineering Research Centre of Cotton, Ministry of Education, College of Agriculture, Xinjiang Agricultural University, 311 Nongda East Road, Urumqi 830052, China

**Keywords:** *Gossypium hirsutum L.*, palmitoyl transferase, *GhPAT* gene family, genome-wide analysis

## Abstract

Protein palmitoylation is an ability of the frame of the cell marker protein is one of the most notable reversible changes after translation. However, studies on protein palmitoylation in cotton have not yet been performed. In our current research, the PAT gene family was systematically identified and bioinformatically analyzed in *G. arboreum*, *G. raimondii*, *G. barbadense* and *G. hirsutum*, and 211 PAT genes were authenticated and classified into six subfamilies. Sixty-nine PAT genes were identified in upland cotton, mainly at the ends of its the 26 chromosomes of upland cotton. The majority of these genes are located in the nucleus of the plant. Gene structure analysis revealed that each member encodes a protein that which contains at least one DHHC structural domain. Cis-acting element analysis indicated that GhPATs genes are mainly involved in hormone production, light response and stress response. Gene expression pattern analysis indicated that most GhPATs genes were differentially expressed upon induction by pathogenic bacteria, drought, salt, hot and cold stresses, and some GhPATs could be induced by multiple abiotic stresses simultaneously. GhPATs genes showed different expression patterns in tissue-specific assays and were found to be preferentially expressed in roots, followed by expression in stems and leaves. Virus-induced gene silencing (VIGS) experiments showed that cotton was significantly less resistant to *Verticillium dahliae* when *GhPAT27* was silenced. We conclude that the *GhPAT27* gene, which mediates S-palmitoylation acetylation, may be involved in the regulation of upland cotton resistance to *Verticillium wilt* (VW). Overall, this work has provided a fundamental framework for understanding the latent capabilities of GhPATs and a solid foundation for molecular breeding and plant pathogen resistance in cotton.

## 1. Introduction

Upland cotton is widely used in the genetic breeding of crops due to its high yield and strong stress resistance [1]. Following the completion of the sequencing of diploid *Gossypium raimondii* and *Gossypium arboreum* [2,3], the genome sequences of the most widely used *Gossypium hirsutum* and *Gossypium barabdense* in production have also been mapped [4]. This marked the beginning of a postgenomic era of functional studies of cotton genes, providing the basis for genome-wide identification and macro-omics analysis of gene families. During plant growth and development, responding to abiotic such as infection by pathogens, and abiotic stress, such as temperature extremes, salt and drought, plants have accumulated multiple mechanisms to cope with resistance to these adverse effects of growth, by prompting them to transmit external stimuli to different cellular compartments in the body [5].

Palmitoyl transferases (PATs) contain transmembrane proteins with 4–6 transmembrane structural domains. The DHHC-CRD conserved structural domain is the active center of the PATs. The cysteine residues are the active sites for the modification function; they are localized on the surface of cytoplasmic lipid droplets and mediate lipid metabolism [6]. Palmitoylation, a posttranslational modification (PTM) that covalently adds the C-16 fatty acyl portion to a specific cysteine, regulates protein localization and protein–protein interactions, and this translational modification has an essential function in animals, plants, and microbial resistance to adversity [7]. In addition, palmitoylation of the DHHC2 C-terminal cysteine, catalyzed by its own PAT activity, regulates the inner membrane complex (IMC) localization of DHHC2 [8]. The IMC-anchored interferon-stimulated protein (ISP) interacts with the microtubule component β-microtubulin to maintain the structural integrity of the subpellicular microtubules (SPM), and the protein palmitoylation cascade binds the cortical membrane with microtubules to maintain the appropriate cytoskeletal structure for syncytial kinetochore differentiation [9].

In recent years, the PAT series of genes has been identified in the genomes of dicotyledonous and monocotyledonous plants to which they belong, such as Arabidopsis, rice and soybean [10,11,12]. For example, in Arabidopsis, *AtPAT10* interacts with the Ca-sensor *AtCBL10* on vesicular plastids for the regulation of salt tolerance [13]; *AtPAT4* affects root hair elongation in plants against external disturbances by mediating membrane binding of the small GTPases ROP2 and actin filament organization [14]; and *AtPAT13* and *AtPAT14* affect S-acylation of NOA1 to regulate nitric oxide signaling and leaf senescence [15]. In rice, the discovery of *OsDHHC30*, which promotes endosomal binding of *OsCBL2* and *OsCBL3* by S-acylation, helps to improve the salt tolerance of the native plant [16]. Detection of *OsDHHC-S-Acylated* protein would be of great help in the interpretation of the functional aspects of the *OsDHHC-OsCBL-OsCIPK* signaling route as well as other pathways in rice [17]. Studies have shown that rice produces a series of hydrogen peroxides when subjected to salt stress, which can limit cell growth and cause oxidative stress. The resistance increases, so it contributes to the salt tolerance of rice seedlings [18]. Additionally, it was also found in other species that genes of the palmitoyl transferase class can regulate the growth process of organisms under stress, and that S-acylation has an instrumental function in vegetative growth and developmental responsiveness to adversity stimulation [19].

The GhPAT family has not yet been investigated. In our study, we used bioinformatics combined with transcriptome expression profiling to systematically identify the GhPAT family members within the genome of cotton based on the sequencing of the cotton genome. We also analyzed the specific locations of GhPAT family genes in subgenome A and subgenome D, the physicochemical properties, subcellular localization, evolution and expression characteristics of GhPAT proteins, and verified the effect of *GhPAT27* on *Verticillium wilt* resistance in upland cotton.

## 2. Results

### 2.1. Identification Information for Members of GhPATs

For the purpose of studying the variability in the copy number of PAT proteins during the evolution of upland cotton, the gene ID, gene name, physical location, molecular weight (MW) and protein length (pI, the pH of the medium at which an amino acid is in a zwitterionic state with zero net charge, expressed in pl.) characteristics of these genes were analyzed. The results showed that 35, 37, 70 and 69 genes were identified in *G. arboreum* (GaPATs), *G. raimondii* (GrPATs), *G. barbadense* (GbPATs) and *G. hirsutum* (GhPATs), respectively. Based on the sequence of gene numbers from the four cotton subspecies, it was determined that the cotton PAT genes are conserved and largely consistent with the architecture of allotetraploid cotton. *GhPAT1* to *GhPAT69* based on their position on the chromosome are shown in Table 1, and the results showed that in *G. hirsutum*, members of the family encode proteins with lengths 433aa (*GhPAT40*) and 509aa (*GhPAT28*), between 49.14 kDa (*GhPAT40*) and 58.27 kDa (*GhPAT28*), and pI ranging from 6.19 (*GhPAT42*) to 10.3 (*GhPAT63*). The theoretical equivalent electric points and relative molecular mass of the products encoded by GhPATs family members do not differ significantly, indicating that the physicochemical properties of GhPATs members’ genes were not significant differences. Of the 69 PAT family genes, 63 PAT proteins had an isoelectric point of more than or equal to 7, demonstrating thar the majority of the amino acids in the PAT proteins were basic.

### 2.2. Chromosomal Distribution of GhPATs

**Figure 1:**
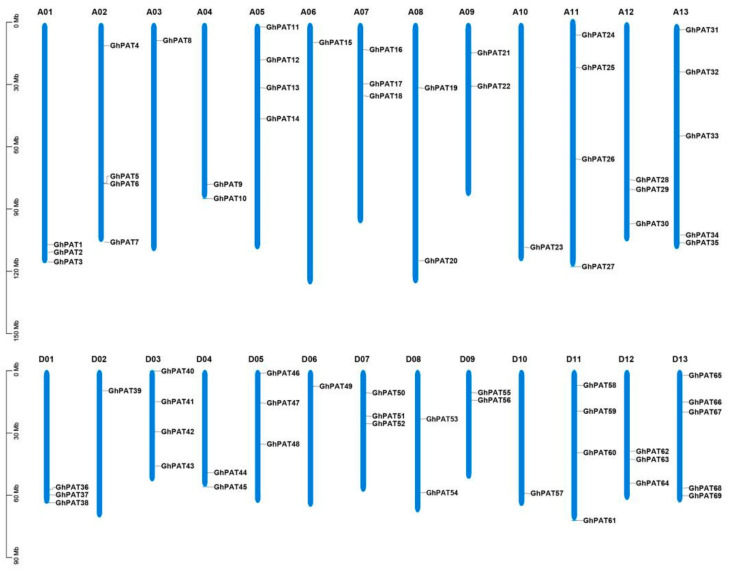
Location of the GhPAT family of genes on the chromosomes.

The chromosome distribution of family members was mapped by TBtools software (v1.098769) using genome gff3 files and gene ID information. The results showed that GhPAT family members were scattered on the chromosome (Figure 1), of which 35 and 34 sequences were contained on subgenomes A and subgenomes D, respectively, and the sequence number of GhPAT genes in subgenome A compared to the sequence for GhPATs in subgenome D, with one PAT gene missing from subgenome D. These results suggested that subgenome D may have lost the gene due to redundancy in gene function during the evolution of cotton; the gene is relatively unevenly distributed on chromosomes, with some chromosomes and chromosomal regions having a high density of gene distribution, and this uneven distribution suggests that genetic variation existed during its evolution.

Using the gene and subgenome distribution of allotetraploid species [20], we found that there are genes located at similar positions on subgenomes A and subgenomes D, then Chr02 and Chr06 of *G. hirsutum*, and that both genes have similar promoter elements, and that *GhPAT4* and *GhPAT39*, *GhPAT15* and *GhPAT49* are homologous in terms of evolutionary relationships. In the GhPAT family, there are 4 sequences on A05 but 3 sequences on D05, and there is one sequence on A03, but 4 sequences on D03. Only one sequence was also found to be located on D02, which contains four sequences; D13 and A13 have the same number of sequences. This result suggests that the GhPATs gene may have been lost or duplicated during the evolutionary process. These results indicated that in the same organism, there is a significant correlation between subgenome A and subgenome D, and this correlation is also the same as the evolutionary process of upland cotton

### 2.3. Phylogenetic Evaluation of GhPATs

**Figure 2:**
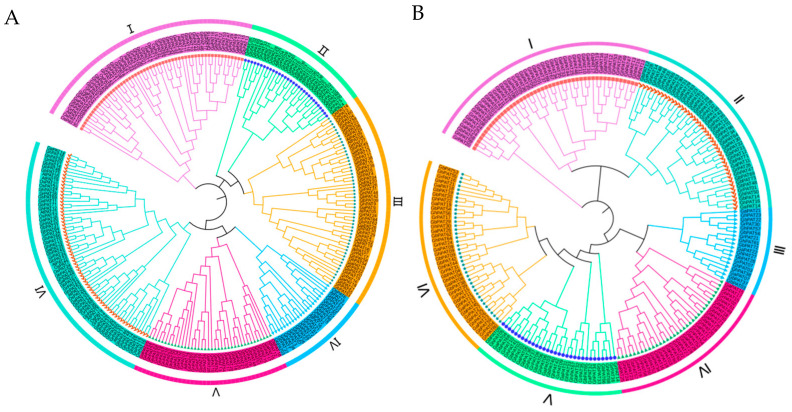
Relationships between phylogenies of PAT family gene members in plants. (**A**) Phylogenetic relationships of PAT family genes in Arabidopsis, rice, *G. arboreum*, *G.* *raimondii*, *G.* *barbadense* and *G. hirsutum*; (**B**) Phylogenetic relationships of PAT genes in *G. arboreum*, *G.* *raimondii*, *G.* *barbadense* and *G. hirsutum*.

To investigate the evolutionary history of PATs in cotton, we constructed a phylogenetic tree of *G. hirsutum* based on the taxonomic relationships of the Arabidopsis and rice PAT protein families using a maximum likelihood (ML) approach with 265 protein sequences (211 sequences in cotton, 24 sequences in Arabidopsis, and 30 sequences in rice) (Table S1). The PAT genes of these plants were divided into six main branches of phylogenetic relationships and were randomly distributed, with evolutionary branch IV having the fewest members (approximately 23 genes), evolutionary branch VI having the most members (approximately 65 genes), and evolutionary branches I, II, III and V containing 57, 31, 53 and 36 genes, respectively. Interestingly, the PAT proteins of Arabidopsis, rice and each of the four types of cotton had corresponding homologs in each evolutionary branch (Figure 2A), which indicated that these PAT proteins in plants were closely related to each other. Heterotetraploid cotton species had essentially twice as many PAT genes per subpopulation as diploid cotton species per subpopulation. Interestingly, the PATs of these four species have corresponding homologs in almost every clade, indicating that the PATs of these species are closely related to each other. The PAT ratio of allotetraploid *G.* *hirsutum* and *G.* *barbadense* was close to 1:1, the PAT ratio of diploid *G.* *raimondii* and *G. arboreum* was also close to 1:1, and the PAT ratio of allotetraploid cotton and diploid cotton was less than 2:1. This may be the result of evolutionary selection during the crossing of two diploid cotton plants to form allotetraploid cotton plants [21].

In a phylogenetic relationship analysis, GhPATs and GbPATs could be clustered on the same branch, a finding that could be used as a basis for gene duplication (Figure 2B). In addition, the combined amount of PAT proteins in tetraploid and diploid cotton confirms that heterotetraploid cotton species are the product of diploid cotton species. Sequence alignment of PAT genes in cotton revealed that most of them with homology were more than 90% identical. An evolutionary tree of PAT family genes in upland cotton was also constructed (Figure 2). The results showed that evolutionary branch I is an ancient PAT genome.

### 2.4. Gene Structural Analysis and Motif Prediction of GhPAT Proteins

Analysis of the gene structure of GhPATs found that genes originating from the same ancestor were distributed in the same branch of the developmental tree, with the number of exons per gene ranging from 4 (*GhPAT 65*, *GhPAT 31*, *GhPAT 48*, *GhPAT 14*, *GhPAT 67*, *GhPAT 33*) to 15 (*GhPAT 65*). In the majority of cases, two genes clustered in the same gene pair were identical in exon-intron structure, such as *GhPAT59/GhPAT25* *and GhPAT69/GhPAT35*. There were also genes that formed gene pairs but had slightly different gene structures and gene lengths, such as *GhPAT55/GhPAT31* and *GhPAT43/GhPAT8*. The number of gene exons varied between evolutionary branches, with 30 genes having no exons, but most members of the same evolutionary branch of GhPATs had the same exon-intron structure. This suggests that GhPAT genes are strongly related to phylogeny on an evolutionary basis and that the gene structure is more complex.

**Figure 3:**
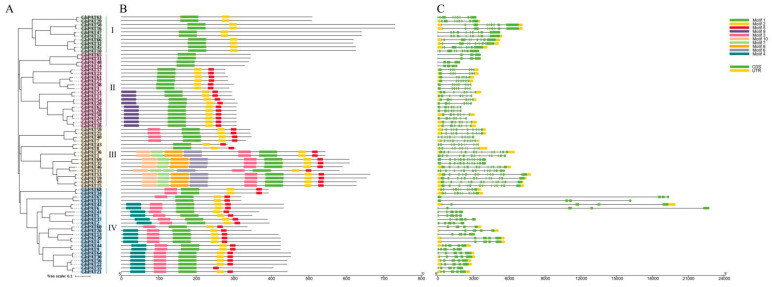
Gene structural analysis and motif prediction of GhPATs proteins (**A**) GhPATs phylogenetic tree; (**B**) GhPATs motif prediction; (**C**) GhPATs inline-exon structure prediction.

Protein sequences of GhPAT members were analyzed using the online tool MEME and 10 motifs in total were obtained. As shown in Figure 3, most of the GhPAT members clustered on the same evolutionary branch and had the same motifs (motif 1, 2 and 5). All GhPAT genes had between 4 and 14 exons, and evolutionary branch III included 8 motif proteins, while GhPAT proteins in evolutionary branch IV lacked motif 2. Interestingly, *GhPAT60* only had motif −3, −1, −2 and −5, whereas the *GhPAT26* gene, which belongs to the same gene pair, included motif −4, −3, −1, −2 and −5. *GhPAT67* had motif 3, −1, −2, −5, −2 and −5, and *GhPAT33*, which is located in the same gene pair, did not have motif 3, so we speculated that *GhPAT60* and *GhPAT67* have lost some of their functions in the evolutionary process (Figure 3). In addition, the clustering of two or more genes into the same gene pair with the same motif indicates that they are involved in similar biological functions at the protein level, and the organization of the more similar exons and introns within the same subgroup further confirms the reliability of the phylogenetic tree.

### 2.5. Structural Domain Analysis and Cis-Acting Element Analysis of GhPATs

**Figure 4:**
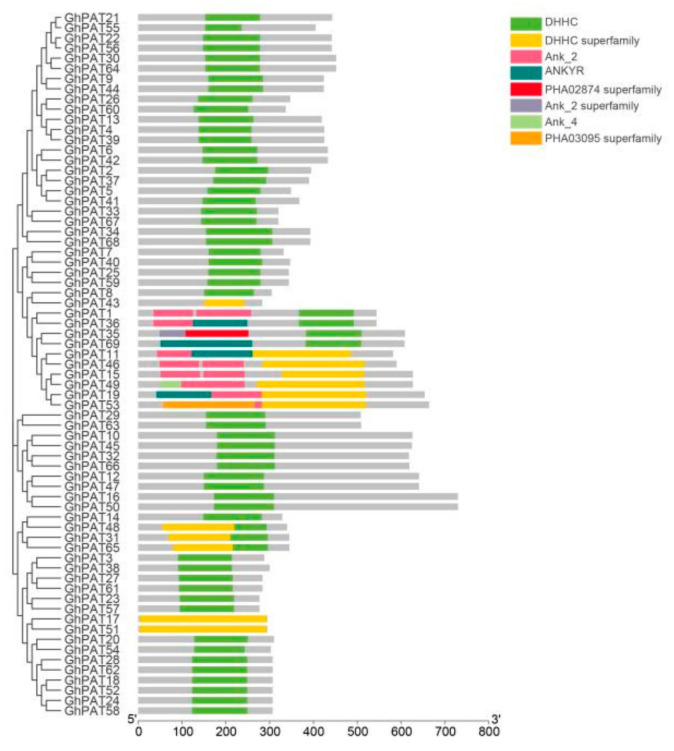
Structural domain analysis of genes that are members of the GhPAT family.

To explore the structural stability of GhPAT genes family member, the conserved structural domains of the genes were analyzed, and the results showed that the GhPAT gene family members contained eight conserved structural domains, of which 69 genes all covered the DHHC and DHHC superfamily conserved structural domains, three GhPAT genes (*GhPAT43*, *GhPAT17* and *GhPAT51*) alone contained DHHC superfamily structural domains, three GhPAT genes (*GhPAT48*, *GhPAT31* and *GhPAT65*) contained only DHHC and DHHC superfamily structural domains, one GhPAT gene (*GhPAT35*) contained Ank-2 superfamily structural domains, and one GhPAT gene (*GhPAT53*) contained the PHA03095 superfamily structural domain (Figure 4). These results suggested that the PAT family gene members are relatively stable in structure.

**Figure 5:**
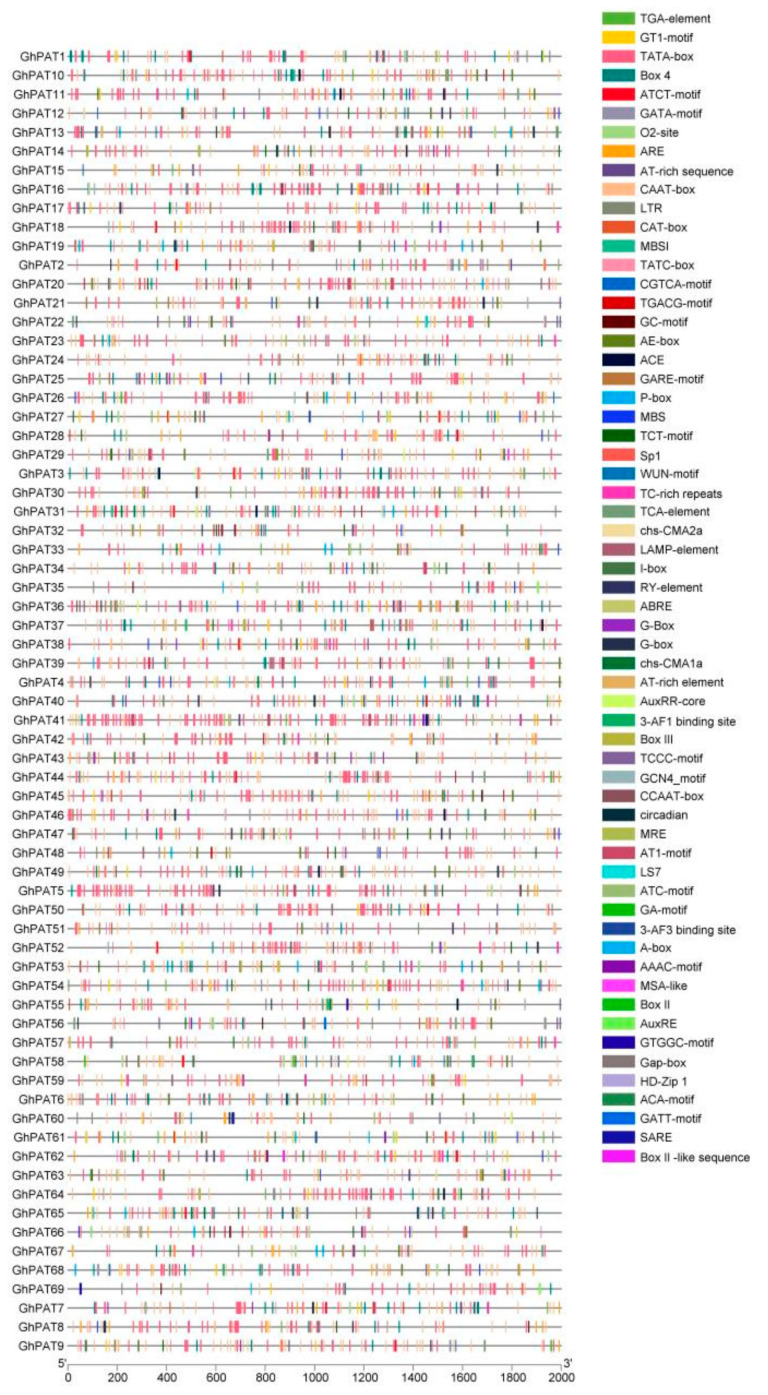
Cis-elements of *GhPAT* genes.

Predicting cis-acting elements can infer the possible functions of proteins encoded by downstream genes, and these elements are distributed differently in different genes (Table S2). These distribution patterns indicate that the induction of light, stress, growth, development, and hormones by these genes can have important effects (Figure 5). Analysis of 69 GhPAT elements showed that there are a large number of core elements in their promoter regions (Figure 5), including TATA-box and light-responsive elements (AE-box, MRE, Sp1, etc.). More cis-responsive elements related to hormones, such as abscisic acid (ABREE), auxin (TGA-element, AuxRR-core), and methyl jasmonate (GA-motif, GARE-motif), were relatively abundant. Most are related to stress, such as drought (MBS, MYB), low temperature (LTR), anaerobic induction (ARE), hypoxia induction and defense and stress response (TC-enriched repeats). There are also cis-elements related to phloem organization (CAT-box, CCGTCC-box, OCT), and some genes contain genes related to zein metabolism (O2-site). This finding fully indicates that GhPATs are involved in the stress response process brought about by plant growth and development and environmental stress.

### 2.6. Covariance Analysis of GhPATs

**Figure 6:**
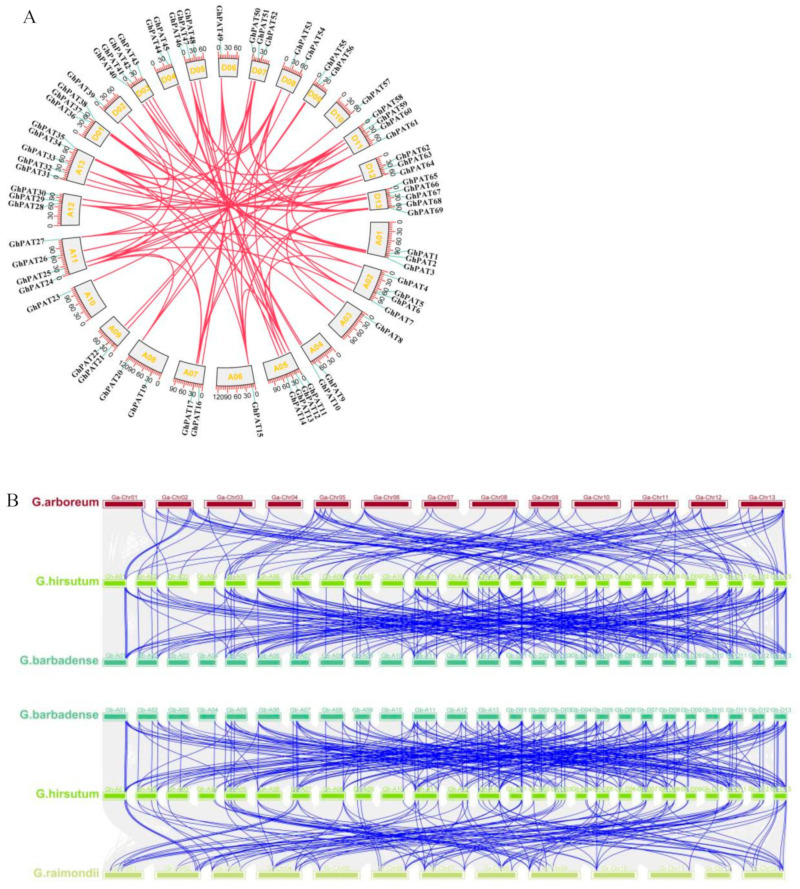
Subgenomic distribution and homozygosity analysis of PAT genes in cotton. (**A**) Intraspecific covariation, synthetic analysis of gene duplicates showing gene pairs between different chromosomes, with gene IDs representing the location of centromeres and the scale on the circles being the megabase; (**B**) Interspecific covariance.

Gene duplication plays an important role in evolutionary mechanism, which can act as a new source of genetic material in genome evolution [22]. *G. hirsutum* and *G.* *barbadense* evolved as a result of crosses between the A genome species and the D genome species. To understand the evolutionary relationship of PAT genes, we produced a map of the relative synthesis of PAT genes in four cotton species. Analysis revealed that several loci were highly conserved between the At subgenomes and Dt subgenomes of the two tetraploid cotton species. First, upland cotton was selected as the core cotton species, and covariates related to other cotton species were constructed (Figure 6). According to MCScan analysis, 104 and 113 orthologs were recognized in diploid cotton species; 77 and 212 orthologs were identified in allotetraploid cotton species. PAT genes on chromosomes D06, D07 and D12 of *G. raimondii* were well covalently related to PAT genes of allotetraploid cotton species. PATs on A11 of *G. arboreum* were well covalently related to PAT genes on homologous chromosomes of allotetraploid cotton species, and GaPATs of A11 also showed collinearity with the PAT genes of allotetraploid cotton species. However, *GhPAT18* was not present during the intraspecific covariance analysis in upland cotton (Figure 6B). Therefore, the results suggest that chromosomal deletions, duplications and tandem repeats may have occurred during the evolution of cotton PAT gene family member genes. In Figure 6B shows that GhAt/GhDt, GbAt/GbDt and multiple chromosomes in subgenomes A and subgenomes D are connected, which means that they are homologous genes, and most PAT genes were retained during the evolution of the polyploid down.

### 2.7. Expression Analysis of GhPATs

**Figure 7:**
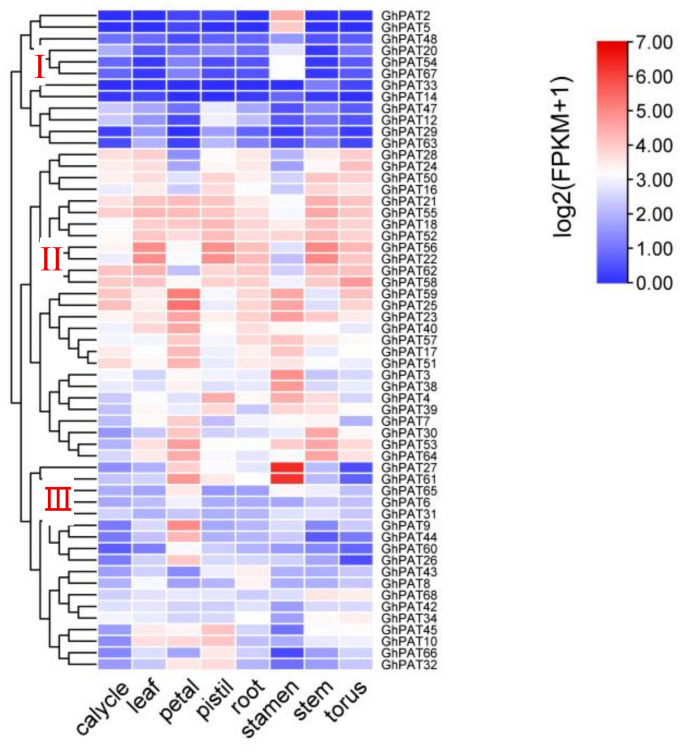
Tissue-specific expression analysis of GhPATs.

To investigate the relationship between gene expression patterns and gene function, various expression patterns of GhPAT genes were detected across different tissues, such as calycle, leaf, petal, pistil, root, stamen, stem, and torus, RNA-seq data (PRJNA248163) downloaded from the NCBI (https://www.ncbi.nlm.nih.gov/sra/?term=PRJNA248163 accessed on 10 August 2022) for upland cotton PAT genes [23]. Sixty-nine GhPATs family genes could be classified into three expression patterns, with each branch having differentially organized expression characteristics (Figure 7). Twelve genes could be classified as the first pattern, with low expression in calycle, leaf and stems and high expression in stamen and stamens; 29 genes were clustered on the second branch, and they were mainly differentially expressed in leaf, roots, stems and torus. The remaining 18 genes were clustered on the third mode of expression, with high variation in leaf floating expression in eight tissues in this mode. There results suggest that the gene expression of members of the GhPAT family of genes is specific and contains more complex functions.

### 2.8. Expression of the PAT Gene Family under Different Stress Conditions in G. hirsutum

**Figure 8:**
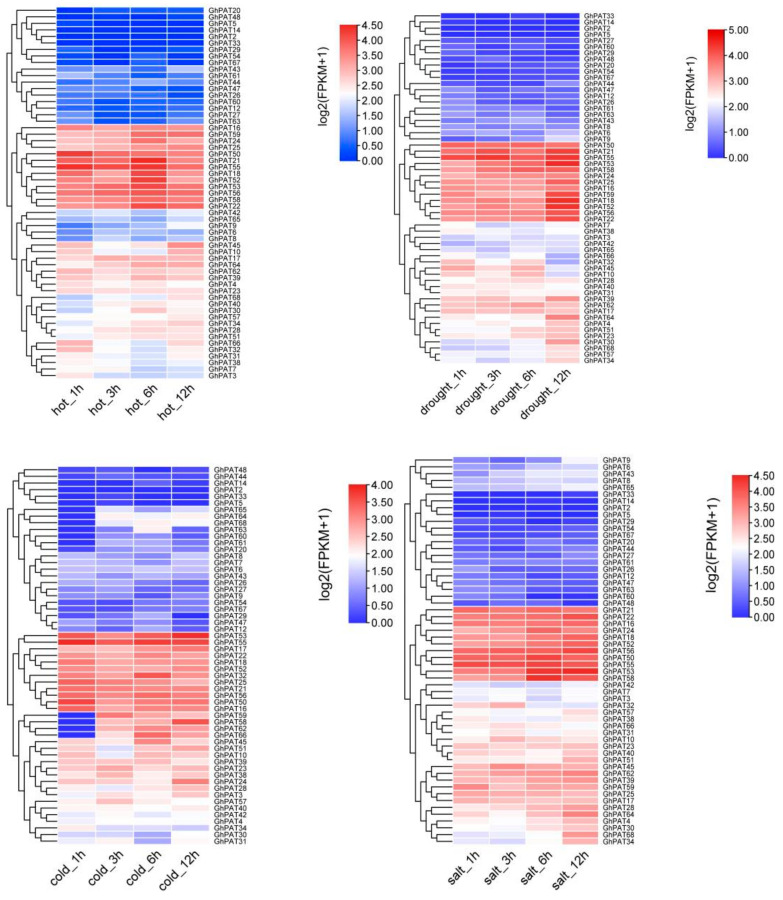
Expression of the GhPAT gene family under different stresses.

To understand the mechanisms of GhPATs response to abiotic stresses, the expression patterns of these genes under cold, heat, salt and polyethylene glycol (PEG) stresses were, and heatmaps were drawn based on expression levels (Figure 8). Some of the GhPAT genes were not significantly differentially expressed under multiple abiotic stresses, and some were strongly induced and significantly differentially expressed under multiple stresses. Under the drought stress of PEG, the expression of 13 genes was upregulated and reached the highest point at 12 h, which shows that 13 genes may be involved in the drought resistance of upland cotton. In a cold environment, 12 genes on the second branch changed significantly (Figure 8). Under hot treatment, 13 genes showed a trend of significantly upregulated expression, but some genes reached the maximum value at 6 h, and began to decline at 12 h. After salt stress treatment, there were 11 genes on the second branch. These 11 genes were the earliest to feel the salt invasion within 6 h and had significant expression, and the expression level continued to be upregulated within 12 h, reaching a maximum at 48 h, and there was continued upregulation of expression. This indicates that the expression trends of cotton PAT genes differed, with the expression of GhPATs obviously increasing under high temperature, salt and PEG stress, which may be of relevance to the engagement of GhPATs in the production of lignin, flavonoids and other phenylpropanoid metabolites. However, under low-temperature, the majority of genes were downregulated, and it was speculated that low temperature might affect the expression of GhPATs. In general, GhPATs in landraces increased with time under high temperature, salt and PEG stress, while they decreased gradually with time under low temperature stress.

In short, 9 genes (*GhPAT21*, *GhPAT22*, *GhPAT18*, *GhPAT16*, *GhPAT55*, *GhPAT50*, *GhPAT53*, *GhPAT52*, *GhPAT56*) were simultaneously highly expressed under abiotic stresses induced by drought, salt, heat and cold. Three genes (*GhPAT21*, *GhPAT24*, *GhPAT58*) showed a trend of high expression under all three stress treatments except for no difference in expression under cold treatment. *GhPAT25* was highly expressed under cold, heat and drought treatments, with no dominant expression under salt stress treatment. This indicates that there is notable variability in gene expression under different stress treatments. Further studies are needed to investigate how PAT genes are regulated under different treatment conditions.

### 2.9. Expression Analysis of GhPATs in Verticillium Dahliae

**Figure 9:**
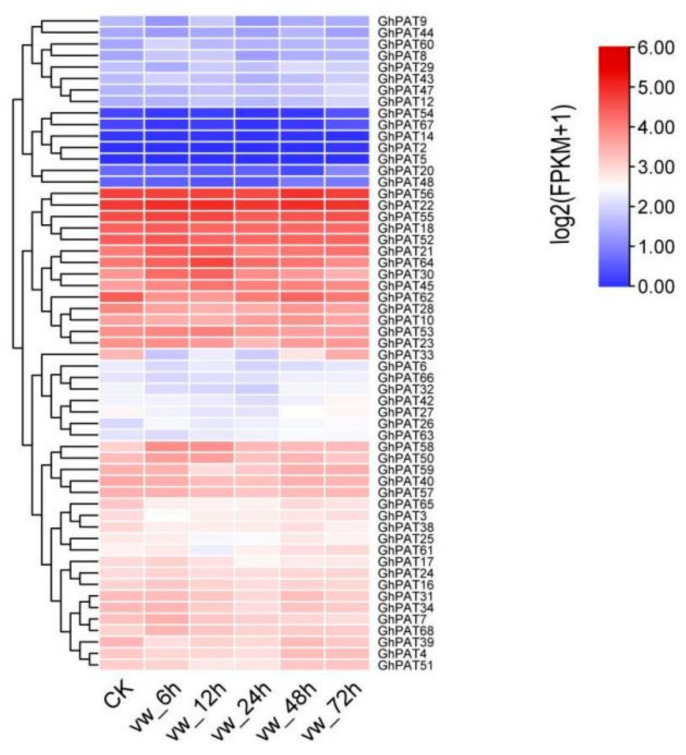
Expression analysis of GhPATs in *V. dahliae*.

To investigate the role of GhPATs in the involvement of *V. dahliae* in biotic stress, RNA-seq data (PRJNA248163) from *G. hirsutum* inoculated with *V. dahliae*, were used for analysis (Figure 9), and the results showed that these GhPAT genes could be aggregated into three expression patterns from 0 to 72 h, with 14 genes showing a high expression trend on the second branch (*GhPAT56*, *GhPAT22*, *GhPAT55*, *GhPAT18*, *GhPAT52*, *GhPAT21*, *GhPAT64*, *GhPAT30*, *GhPAT45*, *GhPAT62*, *GhPAT28*, *GhPAT10*, *GhPAT53*, *and GhPAT23*). Five genes (*GhPAT64*, *GhPAT30*, *GhPAT45*, *GhPAT53* and *GhPAT23*) were found to be expressed at maximum levels at 12 h and then decreased at 24 h and 48 h, which indicated that these genes were engaged in the early defense response of cotton against *V. dahliae.* The expression of *GhPAT27* was also found to be decrease after induction by the pathogen but was characterized by upregulated expression in the subsequent period. Experiments have shown that *GhPAT27* has a functional part in later stages of cotton resistance to infection by *V. dahliae* (Figure 9).

### 2.10. qRT-PCR to Response of GhPAT Genes to V. dahliae

**Figure 10:**
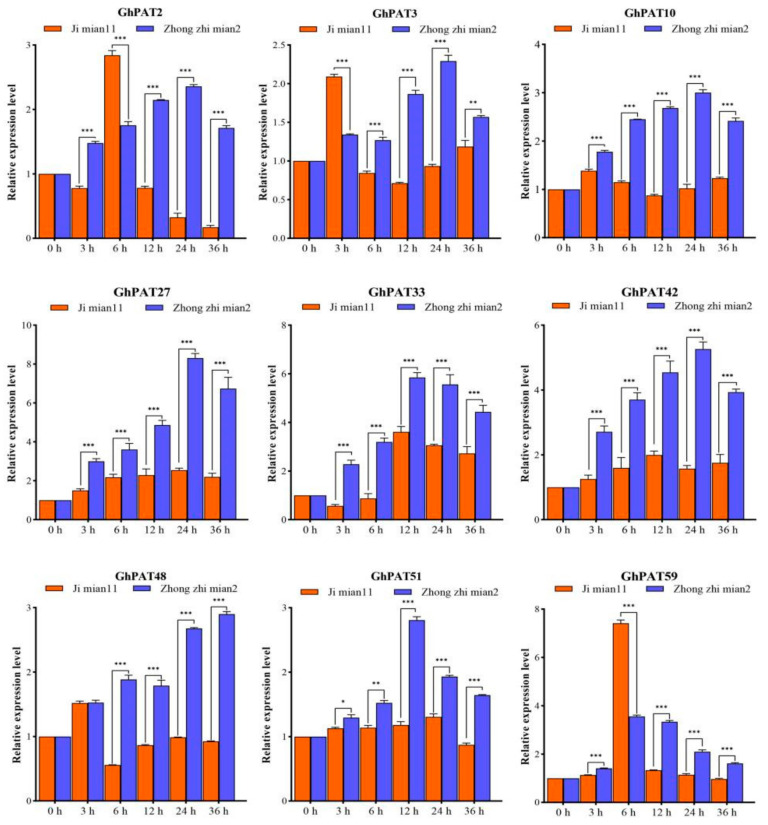
qRT-PCR of the response of GhPATs to *V. dahliae*. The relative expression of *GhPAT27* was all calculated based on the control gene *GhUBQ7*. Three technical replicates and three biological replicates were set up for each experiment. Statistically significant differences from the control group are indicated as * *p* < 0.05; ** *p* < 0.01; *** *p* < 0.001.

Zhong zhimian 2 (resistant cultivar) and Jimian 11 (susceptible cultivar) were used as test materials. According to the upland cotton transcriptome database, the expression patterns of GhPATs were compared. These GhPAT genes were found to change in expression level when induced by *V. dahliae*, and there would be changes in the expression levels, which indicated that GhPATs have a role in the host’s defense against *V. dahliae*. Interestingly, the experimental results of qRT-PCR technology were consistent with the expression pattern of GhPATs in the analysis of transcriptome data. To this end, we postulated that nine genes (*GhPAT2*, *GhPAT3*, *GhPAT10*, *GhPAT48*, *GhPAT51*, *GhPAT59*, *GhPAT27*, *GhPAT33* and *GhPAT42*) are associated with verticillium resistance. To investigate this hypothesis, qRT-PCR was used to analyze the differential expression of these nine GhPATs in disease-resistant and susceptible varieties under pathogenic stress by *V. dahliae*. All but three genes (*GhPAT3*, *GhPAT48* and *GhPAT51*) were found to be significantly altered at different time points after the appearance of the stress response (Figure 10), indicating that these GhPATs genes induced by *V. dahliae* are involved in the parasitization process of *V. dahliae* in cotton. Additionally, six of these genes (*GhPAT2*, *GhPAT10*, *GhPAT59*, *GhPAT27*, *GhPAT33* and *GhPAT42*) were identified and significantly expressed at the same time point. Firstly, we found that the fold change of *GhPAT27* reached 8 by qRT-PCR, while the maximum value of other genes only reached 5, and the expression of other genes at the same time only reached around 3, this indicated that the expression level of *GhPAT27* gene was about three times higher than that of other genes. Secondly, we performed a genome-wide association analysis of data from 300 upland cotton (*Gossypium hirsutum L.*) resources in 10 environments. We found that *GhPAT27* is located on chromosome A11, and this gene can be identified in multiple environmental data associations. Taking everything into account, we predicted that *GhPAT27* might be involved in resistance to verticillium wilt in upland cotton, for which reason we first selected this gene for follow-up work (Figure 10).

**Figure 11:**
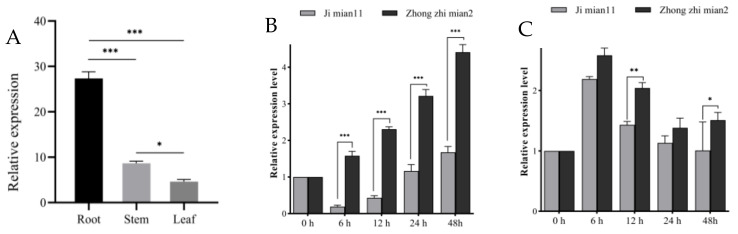
Expression pattern of *GhPAT27*. (**A**) Expression of *GhPAT27* in different tissues; (**B**) Expression level of *GhPAT27* in the roots of Zhong zhimian 2 and Jimian 11; (**C**) Expression level of *GhPAT27* in the stems of Zhong zhimian 2 and Jimian 11. The relative expression of *GhPAT27* was all calculated based on the control gene *GhUBQ7*. Three technical replicates and three biological replicates were set up for each experiment. Statistically significant differences from the control group are indicated as * *p* < 0.05; ** *p* < 0.01; *** *p* < 0.001.

As genes are represented differently in different locations in the plant, they perform different biological functions. To further characterize the function of the predicted gene *GhPAT27*, different materials were inoculated with *V. dahliae* at different times, and tissue-specific expression assays were carried out. When a cotton plant is infected with *V. dahliae*, its root tissue is the first to be sensed, followed by the stem, and finally by the leaves (Figure 11A). In the root tissues, *GhPAT27* transcript levels in Zhong zhimian 2 were significantly higher in both sresistant varieties than in the susceptible variety Jimian 11 at 6 h, 24 h and 48 h after inoculation (Figure 11B). In both disease-resistant and disease-susceptible plants, *GhPAT27* was significantly increased in the stem, and the expression levels of *GhPAT27* were upregulated at 6 h post inoculation and began to decrease at 12 h. However, at 12 h, *GhPAT27* transcript levels were significantly higher in Zhong zhimian 2 than that in Ji mian11 (Figure 11C). Therefore, it is speculated that after the resistant varieties were inoculated with *V. dahliae*, the upregulation of *GhPAT27* expression in the resistant varieties may be one of the reasons for the disease resistance of cotton.

### 2.11. Silencing GhPAT27

**Figure 12:**
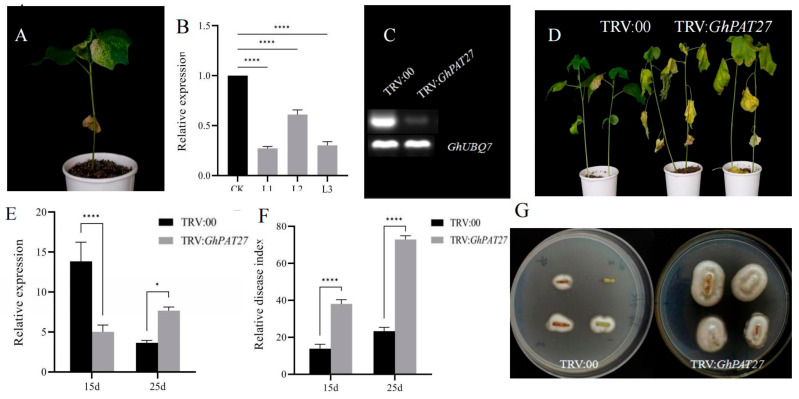
Silencing *GhPAT27* impairs cotton resistance to *V. dahliae.* (**A**) Silenced plants exhibit an albino phenotype. (**B**) Differential expression of *GhPAT27* in silenced and CK plants. CK represents the control; L1, L2, L3 represent three biological replicates, each contains 30 cotton plants. (**C**) RT-PCR analysis confirmed the gene silencing efficiency of TRV:00 and TRV:*GhPAT27* in plant roots. (**D**) Phenotype of *GhPAT27*-silenced plants at 15 d and 25 d. (**E**) Silencing efficiency assay of *GhPAT27* at 15 d and 25 d; (**F**) Relative disease index of *TRV:GhPAT27* at 15 d and 25 d. (**G**) Fungus recovery experiment. Stem sections of TRV:00 and TRV:*GhPAT27* plants were placed on potato glucose agar medium and incubated at 25 °C. The samples were photographed 3 days later. Note: TRV:00 means empty vector; TRV:*GhPAT27* means silenced *GhPAT27* gene. Each contains 30 cotton plants, the relative expression of *TRV:GhPAT27* was all calculated based on the control gene *GhUBQ7*. Statistically significant differences from the control group are indicated as * *p* < 0.05; **** *p* < 0.001. Three technical replicates and three biological replicates were set up for each experiment.

The function of *GhPAT27* was studied using a silencing system in the resistant cultivar Zhong zhimian 2. Approximately 10 days after virus-induced gene silencing (VIGS) injection, the newly grown true chlorophyll in the PDS of the control plant receded, and the phenomenon of whitening gradually appeared, which means that the VIGS carrier was successfully injected into the cotton (Figure 12A). RT-PCR analysis confirmed the gene silencing efficiency of TRV:00 and TRV:*GhPAT27* in plant roots (Figure 12C). The leaves of TRV: GhPAT27 wilted and yellowed after inoculation with the pathogen Vd592 by root immersion, a symptom more severe than that of TRV:00 (Figure 12D). At the same time, approximately 1 g of the leaves from the silenced samples and CK were randomly taken, the gene silencing efficiency of TRV: 00 and TRV: *GhPAT27* plants was detected by RT-qPCR, the results of gene silencing efficiency also showed that the expression level of TRV: *GhPAT27* was significantly lower than that of TRV:00 after silencing (Figure 12E). The disease indices (DI) of TRV:00 and TRV:*GhPAT27* plants were 38.69 and 72.36, respectively (Figure 12D). These results suggested that at 15 days and 25 days after inoculation, the disease index (DI) of TRV:*GhPAT27* were extremely significantly higher than those of TRV: 00 as the time increased (Figure 12F). We conducted recovery tests to assess the extent of *V. dahliae* colonisation of stems and found that fungal growth on stems of TRV:*GhPAT27* plants was significantly more than that on stems of TRV:00 plants (Figure 12G). This result was consistent with the results of qRT-PCR analysis of fungal biomass (Figure 12F). Silencing *GhPAT27* can enhance resistance to *V. dahliae* in upland cotton, and *GhPAT27* may be a positive regulator of plant resistance to pathogen infection. After silencing *GhPAT27*, disruption of the expression of genes related to plant defense.

## 3. Discussion

Proteomics studies have shown that a large number of palmitoylated modified proteins exist in plants, which are engaged in numerous life activities, such as plant growth and development, gamete formation, stress resistance and physiological diseases [24]. Research on the palmitoylation of proteins in plants has been limited, and this understanding is lacking and full of knowledge gaps [25,26].

In this study, 69 PAT palmitoyl transferase genes related to upland cotton were identified in the whole genome. The conserved motifs contained in the PAT gene families of *G. barbadense* and *G. hirsutum* have structures similar to those of the Arabidopsis PAT gene family. Compared with the 24 PAT genes found in Arabidopsis, there are more PAT gene family members in cotton because Arabidopsis is a diploid plant, while *G. barbadense* and *G. hirsutum* are tetraploid [27,28]. The quantitative difference in the DHHC gene family in different species indicates that this gene family has produced a certain degree of differentiation and expansion in the process of plant evolution [29].

GhPAT family gene members have approximately 4 to 15 exons, but some gene members have a higher number of exons, up to 15, and a more complex structure, which may be due to mutation and evolution of the genes over time. This may be due to the mutation and evolution of the gene over time and is indicative of the functional diversity of PAT gene family members [30].

Further construction of phylogenetic trees of upland cotton with Arabidopsis and rice showed that there was no separate division into two categories because of species differences, suggesting that upland cotton is more closely related to the PAT members of Arabidopsis [31]. A mixed phylogenetic tree was constructed by combining with AtPATs. Then, based on genes that have been more intensively studied in the model plants, it is possible to speculate that the more closely related genes in upland cotton, the more clustered the genes in the evolutionary tree, the more closely related they are to each other. The closer the clusters in the evolutionary tree, the more likely they are to have similar functions [32].

Based on gene number, phylogenetic tree and covariance analysis, the GhPAT family of genes can be divided into six subgroups. Sixty-nine GhPATs were found in the upland genome, among which multiple genes of GhPATs gene family members regulate the stress response of cotton to abiotic stress in cotton. Seventy-seven pairs of gene repeats were found in GhPATs, and almost all of them belonged to the duplication of fragment type, suggesting that the expansion of GhPAT genes was mainly achieved through fragment duplication and that the large number of fragment duplication may lead to further sub-functionalization of GhPATs. In addition, the results are consistent and confirmed by the evolutionary analysis among gene family members in other species (such as Arabidopsis and rice) [31,33].

The promoter regions of each GhPAT contain different numbers and types of elements, suggesting that they can participate in different biotic and abiotic stress responses according to different signaling pathways. Among these 69 GhPATs, more than half of the members contain ABA response elements and MeJA response elements involved in the hormone regulation of genes, but only a few genes can be involved in the plant wound response [34]. It is inferred that PAT genes may be take part in the regulation of genes. Various signal responses of plants to improve plant stress resistance. A large number of members of the cotton PAT gene family and similar conserved motifs ensure their functional connection, while differences in gene structure and sequence lead to functional differentiation [35]. At the same time, researchers can study the response mechanism of upland cotton PAT family members to different phytohormone induction and abiotic stresses through promoter analysis [36].

Some NB-LRR disease-resistant proteins, such as R5L1, may function with post-translational modifications via S-acylation of the organism, which can activate downstream functional genes and trigger signaling pathways associated with systemic resistance. Previously, 24 PATs were identified in Arabidopsis that share a conserved DHHC-CRD structural domain, which are involved in palmitoylation modifications of protein substrates and regulate various plant growth or development [37], and data suggested that *AtPAT13* and *AtPAT16* in Arabidopsis achieve enhanced plant resistance to foreign invasion in a functionally additive manner, as a result of S-acetylation of R5L1 mediated. Thus, PAT13 and PAT16 are currently the only PATs—in partnership with the substrate R5L1—known to be involved in disease resistance to *Pseudomonas syringae* *pv* *tomato* *DC3000* (Pst DC3000) in effector-triggered immunity (ETI) at present [38]. We suggest that an in-depth understanding of how S-acylation promotes the immune response mediated by NB-LRR proteins will facilitate research on plant immunity and therefore lead to new strategies for improving the resistance of cotton and other agricultural crops against various pathogens.

## 4. Materials and Methods

### 4.1. Identification and Protein Features

The gene sequences of AtPAT were retrieved from TAIR (Arabidopsis database). The CottonFGD was used to retrieve *G. barbadense* (NAU), *G. hirsutum* (CRI), *G. raimondii* (JGI) and *G. arboreum* (CRI) genome sequence. PAT structural domain sequences were used as template to retrieve possible homologs from the whole genome sequences of *G. barbadense*, *G. hirsutum*, *G. raimondii* and *G. arboreum* via CottonGen’s BLASTP (https://www.cottongen.org accessed on 8 August 2022) [39]. Further information is available through the online tools NCBI CD-Search and Search Pfam to remove incomplete sequences of conserved structural domains and obtain PAT family members [40]. Analysis of the physical properties of proteins encoded by PAT family members by the ExPASy-ProtParam tool [41].

### 4.2. Chromosomal Distribution of GhPATs

*G. hirsutum* reference genome gff3 file and gene ID files were used to map the chromosome positions of family members through MapChart software, the position of GhPATs in the chromosomes of *G. hirsutum* was successfully mapped by the same method. The genome sequences of *G. hirsutum* is downloaded from CottonFGD (https://cottonfgd.org/accessed on 10 August 2022). MapChart software was used to map the chromosomal location of the PAT gene family based on the annotated genomes of *G.* *hirsutum* [42].

### 4.3. Phylogenetic Analysis of GhPATs

Genome annotation files were used to retrieve protein sequence for GhPATs. 265 protein sequences (211 sequences in cotton, 24 sequences in Arabidopsis, and 30 sequences in rice) were used for the phylogenetic tree. PAT protein sequences were aligned and maximum likelihood (ML) phylogenetic trees with 1000 bootstrap replicates were constructed using the MEGA v7.0 program [43]. Both intraspecific and interspecific phylogenetic relationships of PATs were constructed in the same way.

### 4.4. Conserved Motif and Gene Structure Analysis of GhPATs

For GhPATs, we used the MEME (http://meme-suite.org/accessed on 1 August 2022) website to identify the conserved sequence of the protein. The maximum motif parameter of the gene was 10, the rest of the parameters remain unchanged, and the domain files of GhPATs were obtained accordingly [44]. The gene evolution relationship file and gff3 file downloaded from the MEME website and CottonFGD, and based on them to plot the conserved motif and gene structure of GhPAT family members with TBtools software (v1.098769) [45].

### 4.5. Structural Domain and Cis-Acting Element Analysis of GhPATs

The required information of the upland cotton PAT family genes was extracted from the cotton genome database CottonFGD (https://cottonfgd.org/accessed on 10 August 2022), and the obtained sequences were then submitted to the Plant CARE database to identify possible sequences in the promoter region. formula action element [45,46].

### 4.6. Covariance Analysis of GhPATs

MCScan toolkit (V1.1) was used to examine gene duplication events in the PAT family in upland cotton [47], and Tbtools software was used to extract and display gene duplication events in PAT family members.

### 4.7. Expression Analysis of GhPATs in Cotton under Different Tissues and Stresses

Transcriptome data for eight tissues of upland cotton, including root, stem, leaf, pistil, stamen, calyx, petal and receptacle (PRJNA248163), as well as transcriptome data for four stress types (cold, hot, drought and salt stress) were downloaded [48]. The cotton material processing and data acquisition process are detailed in the research report [49]. Using FPKM > 1 as the screening standard for gene expression, log2(FPKM + 1) normalization of transcriptome data was performed. The expression heatmap of PAT family members was drawn using TBtools software (v1.098769) [45].

### 4.8. Analysis of the Disease Resistance Expression Pattern of GhPATs

According to the RNA-seq data, the differentially expressed genes of upland cotton under the stress of *Verticillium dahliae* were analyzed. Genes with stress response to *Verticillium* dahliae in members of the GhPATs family were screened, and the heatmap, phylogenetic tree and cis-regulatory element were generated by the number of fragments per kilobase exon (FPKM) using TBtool software. The GhPAT family members were classified according to their expression at different times, and the expression pattern of the GhPAT family members at different times of resistance to *V. dahliae* was investigated. Heatmaps were drawn using TBtools software (v1.098769) for hierarchical cluster analysis.

### 4.9. RNA Extraction and qRT-PCR

Zhong zhimian 2 and Jimian 11 in the test materials were planted in a cotton cultivation room according to conventional cultivation methods, and water was supplied every 2 days [50]. The lower middle part of the pot was cut with a razor blade when the plants were two-leafed, ensuring that the roots were damaged, and the plants were treated with a spore suspension concentration of 2.8 × 10^8^ mol/L of the reserve solution Vd592 (Vd592 is a highly pathogenic deciduous strain of *V. dahliae*) that was injected into the seedling bags. The roots and steam tissue were collected at 0 h, 3 h, 6 h, 12 h, 24 h and 36 h, and placed in liquid nitrogen. Samples were extracted using the RNA Prep Pure Plant Plus Kit extraction kit, and the first cDNA strand was synthesized using the All-in-One First-Strand cDNA Synthesis Super Mix for qPCR Kit (One-Step gDNA Removal) (TransGen, Beijing, China). Based on the cDNA information of the GhPATs gene, primers for specific regions of the gene sequence were designed using Primer 5.0 software at the 5′ or 3′ end of the gene sequence (Table S3). Using cDNA obtained from root tissue as a template and *GhUBQ7* (GenBank: No. AY189972) as an internal reference gene, a 20µL reaction system was used with the following components: 2 µL (200 ng) cDNA, 0.4 µL forward primer (10.0 µmol/L), 0.4 µL reverse primer (10.0 µmol/L), 10 µL 2 × TransStart Top/Tip Green qPCR Super Mix and 7.2µL ddH_2_O. The reactions were completed according to the following procedure: 94 °C for 30 s; 94 °C for 5 s, 60 °C for 15 s, 72 °C for 10 s, 45 cycles; and end at 4 °C. The device used for qRT-PCR was Applied Biosystems@7500 fast (Applied Biosystems is an excellent product developed by Thermo Fisher Scientific Life Science Products, originally from Singapore, under the brand name Applied Biosystems, model 7500 Fast Real-Time PCR System), and relative expression levels were calculated according to the 2^−ΔΔCT^ method. Three technical replicates and three biological replicates were set up for each experiment.

### 4.10. Silencing the Target Gene GhPAT27

We used Virus-induced gene silencing (VIGS) to examine whether *GhPAT27* plays a role in upland cotton resistance to *V. dahliae*. Test strains and test vectors: the Vd592 (Vd592 is a highly pathogenic deciduous strain of *V. dahliae*) strain, the agrobacterium strain GV3101, the TRV virus vector and the agrobacterium strain containing the positive control vector were all kept in our laboratory, and the E. coli receptor cells ‘Trans-T1’ were purchased from Beijing Quanshijin Biotechnology Co., Ltd. (Beijing, China) *GhPAT27* CDS was integrated into vector TRV:*GhPAT27*, a specific *GhPAT27* gene fragment was inserted into the restriction site of silencing vector TRV:*GhPAT27*, the silencing vector TRV:*GhPAT27* was constructed, and TRV:*GhPAT27* was transformed into GV3101(agrobacterium tumefaciens strain) [51]. First, Zhong zhimian 2, a resistant variety planted during planting, when the two cotyledons of the seedling were fully unfolded, a needle of a disposable 1 mL syringe was used to make two small wounds on the lower epidermis of the cotton cotyledon that had just been flattened. TRV:00 and TRV:*GhPAT27* were injected into the abaxial surface of newly emerged cotyledons of seedlings. (Figure 12). When injecting, even force was applied so that the bacterial liquid filled the entire leaf. Cultures were performed under light/8 h and dark/16 h conditions. When the positive control plants showed an albino phenotype, the true leaves of the empty vector cotton and the true leaves of the plants that silenced the target gene were randomly selected, using cDNA obtained from root tissue as a template, and *GhUBQ7* (GenBank: No. AY189972) as an internal reference gene, to detect the gene silencing efficiency by qRT-PCR, primers for specific regions of the gene sequence were designed using Primer 5.0 software at the 5′ or 3′ end of the gene sequence (Table S3). The relative expression of TRV:*GhPAT27* was all calculated based on the control gene *GhUBQ7*. Three technical replicates and three biological replicates were set up for each experiment(Figure 12). Chapella medium was prepared, Vd592 (Vd592 is a highly pathogenic deciduous strain of *V. dahliae*) was cultivated, and a spore suspension was made at a concentration of 2.8 × 10^8^ mol/mL. After the positive plants exhibited an albino phenotype, the cotton-silenced plants and empty control plants were infected and inoculated, and 50 mL of *V. dahliae* was injected into each nutrient bowl (Figure 12). The leaves of the silenced plants and CK plants at 15 d and 25 d after infection with Vd592 were collected, total RNA was extracted, and cDNA was obtained by reverse transcription. The content of *V. dahliae* in plant leaves was quantified by qRT-PCR with *V. dahliae* internal reference gene primers.

## 5. Conclusions

The characteristics of GhPAT members in upland cotton were identified and analyzed at the genome-wide level, and it was found that the unexpected hybridization and genome-wide doubling events of diploid cotton species promoted the expansion of the family. The GhPAT gene family was structurally conserved in themselves and in the encoded products during evolution and were subjected to purifying selection. Virus-induced gene silencing (VIGS) and disease resistance analysis showed that silencing *GhPAT27* made cotton more susceptible to the disease, and further study revealed that silencing *GhPAT27* could lead to the spread of *V. dahliae* in cotton, a large number of pathogens can break through the cell wall and colonize the plant. In conclusion, the *GhPAT27* gene can regulate cotton to *V. dahliae* and can be used as a candidate gene for disease resistance breeding. This study broadens our understanding of the role of palm acyltransferase in resistance to verticillium wilt in upland cotton and provides an important scientific basis for developing strategies to control verticillium wilt.

## Figures and Tables

**Table 1:** Identification information for members of the GhPAT gene family.

Gene ID	Gene Name	Genomic Location	Protein Length (bp)	Molecular Weight (Da)	Theoretical Isoelectric Point (pI)
Gh_A01G201000	*GhPAT1*	A01: 106,644,168–106,649,891	544	60,329.10	7.67
Gh_A01G217400	*GhPAT2*	A01: 110,355,592–110,357,821	395	44,677.10	8.13
Gh_A01G248800	*GhPAT3*	A01: 114,993,971–114,996,727	288	32,621.46	8.74
Gh_A02G074800	*GhPAT4*	A02: 10,783,870–10,789,492	425	48,254.43	7.95
Gh_A02G139500	*GhPAT5*	A02: 77,013,588–77,015,623	349	39,891.37	9.09
Gh_A02G139600	*GhPAT6*	A02: 77,326,344–77,349,111	433	49,247.55	6.5
Gh_A02G207100	*GhPAT7*	A02: 105,408,865–105,412,345	332	38,266.80	8.56
Gh_A03G054400	*GhPAT8*	A03: 8,316,187–8,320,341	305	34,420.65	8.48
Gh_A04G122000	*GhPAT9*	A04: 77,576,070–77,578,084	424	48,357.21	8.69
Gh_A04G170800	*GhPAT10*	A04: 84,471,911–84,475,376	626	68,684.92	8.59
Gh_A05G010000	*GhPAT11*	A05: 1,406,399–1,412,030	582	64,090.24	7.09
Gh_A05G162000	*GhPAT12*	A05: 17,248,575–17,253,983	641	70,277.50	8.38
Gh_A05G258700	*GhPAT13*	A05: 30,687,878–30,690,980	419	47,780.74	7.48
Gh_A05G301400	*GhPAT14*	A05: 45,644,709–45,646,354	329	36,863.26	8.93
Gh_A06G057800	*GhPAT15*	A06: 9,253,637–9,260,874	627	69,025.32	6.71
Gh_A07G090000	*GhPAT16*	A07: 12,680,862–12,687,974	730	79,039.88	8.56
Gh_A07G144800	*GhPAT17*	A07: 29,190,890–29,193,768	295	33,122.02	8.21
Gh_A07G158400	*GhPAT18*	A07: 35,012,548–35,015,765	307	34,645.40	7.69
Gh_A08G096600	*GhPAT19*	A08: 31,121,482–31,128,915	654	71,782.47	7.21
Gh_A08G198400	*GhPAT20*	A08: 114,519,379–114,522,238	310	34,997.23	8.61
Gh_A09G042400	*GhPAT21*	A09: 14,285,815–14,288,513	443	49,732.03	8.41
Gh_A09G053600	*GhPAT22*	A09: 30,324,111–30,326,963	442	49,694.26	8.53
Gh_A10G209400	*GhPAT23*	A10: 107,898,560–107,901,981	277	31,495.96	8.75
Gh_A11G086800	*GhPAT24*	A11: 7,557,651–7,560,097	307	34,754.33	7.42
Gh_A11G181600	*GhPAT25*	A11: 23,238,492–23,242,550	344	39,451.13	8.17
Gh_A11G240600	*GhPAT26*	A11: 67,374,084–67,379,186	347	39,639.16	8.57
Gh_A11G375800	*GhPAT27*	A11: 119,164,180–119,167,139	284	31,907.41	8.53
Gh_A12G117500	*GhPAT28*	A12: 75,796,826–75,798,806	307	34,960.57	6.59
Gh_A12G137100	*GhPAT29*	A12: 80,379,332–80,382,877	508	58,141.21	10.27
Gh_A12G216400	*GhPAT30*	A12: 96,932,476–96,935,605	452	51,452.17	8.41
Gh_A13G025000	GhPAT31	A13: 2,645,301–2,648,903	345	38,583.93	8.94
Gh_A13G084800	*GhPAT32*	A13: 22,978,902–22,984,011	618	68,051.95	8.51
Gh_A13G107100	*GhPAT33*	A13: 53,794,071–53,810,304	320	36,354.90	8.54
Gh_A13G196000	*GhPAT34*	A13: 101,505,059–101,508,851	393	44,823.33	9.13
Gh_A13G228400	*GhPAT35*	A13: 105,162,566–105,166,602	609	66,921.37	7.43
Gh_D01G198500	*GhPAT36*	D01: 57,318,599–57,325,051	544	60,259.05	7.67
Gh_D01G213700	*GhPAT37*	D01: 59,934,237–59,937,432	390	44,173.59	8.33
Gh_D01G243100	*GhPAT38*	D01: 63,762,802–63,765,621	300	34,025.02	9.28
Gh_D02G077600	*GhPAT39*	D02: 9,776,929–9,782,516	425	48,308.45	7.99
Gh_D03G003900	*GhPAT40*	D03: 257,592–260,644	347	39,868.76	8.2
Gh_D03G067200	*GhPAT41*	D03: 15,062,867–15,064,986	368	42,022.34	8.93
Gh_D03G086100	*GhPAT42*	D03: 29,483,457–29,503,379	433	49,139.33	6.19
Gh_D03G142600	*GhPAT43*	D03: 46,045,864–46,049,299	283	32,108.88	7.85
Gh_D04G161600	*GhPAT44*	D04: 49,275,947–49,278,715	424	48,386.27	8.9
Gh_D04G217000	*GhPAT45*	D04: 56,134,857–56,139,025	625	68,509.73	8.59
Gh_D05G014000	*GhPAT46*	D05: 1,289,795–1,295,927	590	65,581.87	7.02
Gh_D05G179000	*GhPAT47*	D05: 15,635,522–15,640,760	641	70,308.72	8.26
Gh_D05G310400	*GhPAT48*	D05: 35,421,295–35,423,177	340	37,957.65	8.65
Gh_D06G056100	*GhPAT49*	D06: 7,676,920–7,684,097	627	69,085.33	6.74
Gh_D07G088700	*GhPAT50*	D07: 10,795,175–10,802,285	730	78,955.76	8.39
Gh_D07G143900	*GhPAT51*	D07: 21,950,634–21,954,273	295	32,994.85	8.3
Gh_D07G157400	*GhPAT52*	D07: 25,658,584–25,661,866	307	34,660.37	7.69
Gh_D08G098900	*GhPAT53*	D08: 23,438,004–23,445,817	664	72,891.51	7.19
Gh_D08G194900	*GhPAT54*	D08: 58,917,795–58,921,035	303	34,398.53	8.47
Gh_D09G037600	*GhPAT55*	D09: 10,675,800–10,677,929	405	45,321.87	8.42
Gh_D09G045200	*GhPAT56*	D09: 10,675,800–10,677,929	442	49,607.18	8.47
Gh_D10G232300	*GhPAT57*	D10: 59,176,702–59,180,114	277	31,577.09	8.75
Gh_D11G087400	*GhPAT58*	D11: 7,193,621–7,196,712	307	34,806.38	7.42
Gh_D11G183900	*GhPAT59*	D11: 19,600,308–19,604,330	344	39,533.21	8.09
Gh_D11G241300	*GhPAT60*	D11: 39,646,391–39,650,033	337	38,582.85	8.37
Gh_D11G383600	*GhPAT61*	D11: 72,301,279–72,304,312	284	31,960.38	8.53
Gh_D12G117600	*GhPAT62*	D12: 39,003,340–39,005,322	307	35,016.57	6.41
Gh_D12G137300	*GhPAT63*	D12: 42,848,700–42,851,950	509	58,398.57	10.3
Gh_D12G209200	*GhPAT64*	D12: 54,340,099–54,343,145	452	51,129.85	8.64
Gh_D13G026800	*GhPAT65*	D13: 2,399,443–2,403,053	345	38,584.87	8.69
Gh_D13G087200	*GhPAT66*	D13: 15,254,636–15,259,872	619	68,219.12	8.47
Gh_D13G098700	*GhPAT67*	D13: 20,048,482–20,067,879	320	36,331.89	8.47
Gh_D13G198500	*GhPAT68*	D13: 56,689,778–56,693,401	393	44,679.16	8.97
Gh_D13G232500	*GhPAT69*	D13: 60,344,782–60,348,822	608	67,203.66	7.43

## Data Availability

Not applicable.

## Supplementary Materials

The following supporting information can be downloaded at: https://www.mdpi.com/article/10.3390/plants11202758/s1, Table S1: PAT protein sequence; Table S2: Cis-acting element; Table S3: Primer sequences used in this study.
